# Novel Regulatory Factors in the Hypothalamic-Pituitary-Ovarian Axis of Hens at Four Developmental Stages

**DOI:** 10.3389/fgene.2020.591672

**Published:** 2020-11-04

**Authors:** Jing Li, Chong Li, Qi Li, Guoxi Li, Wenting Li, Hong Li, Xiangtao Kang, Yadong Tian

**Affiliations:** College of Animal Science and Veterinary Medicine, Henan Agricultural University, Zhengzhou, China

**Keywords:** reproductive hormones, HPO axis, DEGs, regulatory factors, hens

## Abstract

Ovarian follicular development is an extremely complex and precise process in which the hypothalamic-pituitary-ovarian (HPO) axis plays a crucial role. However, research on the regulatory factors of the HPO axis is sparse. In this study, transcriptomes of the tissues in the entire HPO axis at 15, 20, 30, and 68 w of age were analyzed. In total, 381, 622, and 1090 differentially expressed genes (DEGs) were found among the hypothalamus, pituitary, and ovary, respectively. In particular, the greatest number of DEGs (867) was identified from the comparison of ovary at 30 and 15 w, which might be related to ovarian development and function at high ovulation capacity. The Gene Ontology (GO) and Kyoto Encyclopedia of Genes and Genomes (KEGG) analyses indicated that most of these DEGs in the significantly enriched biological process (BP) terms and pathways were primarily involved in tissue development and the regulation of reproductive hormone biosynthesis and secretion. The latter is highly related to the HPO axis. Therefore, a number of hub candidate genes strongly associated with the HPO axis in each tissue were filtered by analyzing the Protein-protein interaction (PPI) network and seven known reproductive hormone-associated key genes were obtained: *PGR, HSD3B2, CYP17A1, CYP11A1, CYP21A2, STS*, and *CYP19A1*, and 12 novel genes: *ROCK2, TBP, GTF2H2, GTF2B, DHCR24, DHCR7, FDFT1, LSS, SQLE, MSMO1, CYP51A1*, and *PANK3*. These will be utilized for further research into the function of the HPO axis. This study has highlighted the major role of the HPO axis in the reproduction of hens at the four developmental stages and explored the novel factors that might regulate reproduction, thus providing new insights into the function of the HPO axis on the reproductive system.

## Introduction

Egg production is an essential trait for laying hens and for breeding in poultry. The growth and development of chicken ovarian follicles has been found to be directly related to the egg-laying performance (Williams and Sharp, 1978b). Unlike mammals, chicken gonads are asymmetric in the embryonic stage, with only the left ovary functioning and the right ovary degenerating during development (Caicedo Rivas et al., 2016; Yu et al., 2017). In general, a hen’s ovary begins to grow at 14 w after hatching, and the primordial follicles transition to secondary follicles. Ovary weight increases exponentially and gradually generates pre-hierarchical follicles before sexual maturity (around 18–20 weeks of age) (Robinson et al., 2001). After the first oviposition, the chicken ovary continues to develop to its largest size until reaching peak egg production. The ovary contains multiple follicles of various sizes and developmental stages during the active laying period to maintain ovulation and produce eggs. After the peak period, the number of viable follicles declines gradually and the proportion of atretic follicles increases, leading to a reduction in egg production (Waddington et al., 1985; Lillpers and Wilhelmson, 1993).

In laying hens, ovarian follicular development is an extremely complex and precise process involving pre-hierarchical follicle recruitment, selection, and dominance, pre-ovulatory follicles, and ovulation (Johnson, 2015). There are multiple internal factors that affect the follicular development of hens, such as hormones, cytokines, regulators, genes, and proteins, which are linked to a variety of signaling pathways (Onagbesan et al., 2000; Sundaresan et al., 2008; Caicedo Rivas et al., 2016; Nowak et al., 2017). Many researchers have focused on the role of the hypothalamic-pituitary-ovarian (HPO) axis in the process of reproduction, including follicle development and differentiation, ovulation, and follicular atresia (Johnson et al., 1996). The HPO axis can regulate the ovulation cycle and egg production directly or indirectly by mediating the levels of reproductive hormones secreted by the hypothalamus, pituitary, and ovary (Komatsu and Masubuchi, 2017; Brady et al., 2020). Gonadotropin-releasing hormone (GnRH) can stimulate the secretion of follicle stimulating hormone (FSH) and luteinizing hormone (LH) from the anterior pituitary gland, subsequently triggering the release of estradiol, androgen, and progesterone in ovarian follicles. These hormones can also inhibit the release of GnRH and gonadotropin hormones, via negative feedback, to maintain the homeostasis of reproductive hormones (Williams and Sharp, 1978a; Padmanabhan et al., 2002). Moreover, other hormones and neuropeptides, such as gonadotropin-inhibitory hormone (GnIH), growth hormone (GH), and prolactin secreted by the hypothalamus and pituitary, are also involved in the regulation of ovarian follicular development and reproduction by the HPO axis (Bédécarrats et al., 2009; Hrabia et al., 2011; Hrabia, 2015). Some researchers have reported that the secretion of reproductive hormones varies with age in chickens, and the majority of the sex hormones are generated at different stages of ovarian follicle growth (Biswas et al., 2010; Brady et al., 2020). However, an improved understanding of the fundamental regulatory mechanisms controlling the HPO axis is still required.

In recent years, the rapid advancement in molecular biotechnology has been utilized to carry out research into the molecular mechanisms. Hence, to explore the novel genes and regulatory mechanisms of the HPO axis during different stages of ovarian development in hens, the transcriptomes of whole HPO tissues (hypothalamus, pituitary, and ovary) were analyzed by RNA-seq during four stages: initial ovarian development, sexual maturation, the peak and late laying periods. This study will provide new insights into the function of the HPO axis at different developmental stages of the ovary during reproduction.

## Materials and Methods

### Ethics Approval

All animal experiments and animal care in this study were performed in accordance with the regulations for the administration of affairs concerning experimental animals (Revised Edition, 2017). The protocols were approved by the Henan Agricultural University Institutional Animal Care and Use Committee (Permit Number: 19-0068).

### Experimental Design, Animals, and Management

A total of 36 healthy Hy-line brown laying hens at 15, 20, 30, and 68 weeks of age were provided by the poultry germplasm resource farm of Henan Agricultural University and were housed in separate cages, in which each age phase consisted of 9 hens. Commercial feed and clean drinking water were supplied *ad libitum*. The procedures of photoperiod and immunity were performed in accordance with the regular farm management. Selected hens were anesthetized by intravenous injection of pentobarbital sodium (3%, 30 mg/kg body weight) in the wing vein. Under deep anesthesia, hens were euthanized via jugular vein bleeding for sample collection. For sample preparation, the tissues of hypothalamus and pituitary were extracted from each individual and the whole tissue of ovary was collected after removing the follicles that were larger than 2 mm in diameter, then all tissues were immediately frozen in liquid nitrogen and stored at −80°C for RNA extraction.

### Sample Preparation, RNA Isolation, and Sequencing

Total RNA was isolated from 36 tissues samples (12 hypothalamus, 12 pituitary, and 12 ovary samples) using Trizol RNA extraction reagent (Invitrogen, Carlsbad, CA, United States), with each sample consisting of a homogenous mixture from three hens to reduce individual variation. The purity, concentration, and integrity of the RNA were measured and checked using the Nano Photometer spectrophotometer (IMPLEN, CA, United States), Qubit RNA Assay Kit in Qubit 2.0 Fluorometer (Life Technologies, CA, United States), and an RNA Nano 6000 assay kit of the Agilent Bioanalyzer 2100 system (Agilent Technologies, CA, United States), respectively. All cDNA libraries were sequenced using an Illumina Hiseq 2500 platform and 125 bp paired-end reads were generated.

### Quality Control

Raw data (raw reads) in the fastq format were first processed through in-house Perl scripts. Clean data (clean reads) were screened by removing reads containing adapters, reads containing ploy-N, and low-quality reads from the raw data. At the same time, Q20, Q30, and GC content of the clean data were calculated. All the downstream analyses were based on the clean data of high quality.

### Quantification of Transcripts and Differentially Expressed Gene Analysis

FPKMs (Fragments per kilo-base of exon per million fragments mapped) of gene expression levels in each sample were calculated using StringTie (v2.1.1) and the obtained read counts were normalized by TMM (Pertea et al., 2016). Differentially expressed gene (DEG) profiles of the three tissues at the four stages were analyzed using the edgeR package (R-3.2.4), and empirical Bayes was used to moderate the degree of overexpression across the transcripts (Robinson et al., 2010). Significant DEGs were identified with adjusted *p* < 0.05, | log fold chang | ≥ 1, and an FPKM > 1 in at least one group.

### Hierarchical Clustering and Annotation Analysis

Hierarchical cluster analysis of the DEGs filtered from the three tissues at different weeks of age was performed using g-plots in the R package (Walter et al., 2015). The GO enrichment analysis of the DEGs was performed using the GOseq R package (Young et al., 2010). A *p*-value < 0.01 was used to indicate the significantly enriched terms. Pathway enrichment analysis was implemented using the KEGG database and KOBAS software programs to test the statistical enrichment of the DEGs (Kanehisa et al., 2008). A *p*-value < 0.05 was used to indicate a significantly enriched pathway.

### Protein-Protein Interaction Analysis

The DEGs associated with the regulation of the HPO axis on ovarian function were extracted according to the combinations of the GO and KEGG analyses. All protein interaction predictions for these DEGs were performed using the STRING database (Von Mering et al., 2003), and all interactions were filtered with a minimum confidence score of 0.7. The Protein-Protein Interaction (PPI) network diagram was plotted using Cytoscape (v3.7.2) (Cline et al., 2007).

### Validation of RNA-Seq Data by Quantitative Real Time PCR (qRT-PCR)

RNA expression was detected using a Primer Script RT reagent Kit (TaKaRa, Dalian, China), according to the manufacturer’s protocol. Real-time PCR was performed using iTaqTM Universal SYBR Green Supermix Kit (Bio-Rad Laboratories Inc., Waltham, MA, United States) using a Light Cycler 96 instrument (Roche, Basel, Switzerland) at 95°C for 3 min; 40 cycles of 95°C for 10 s, annealing at 60°C for 30 s, 72°C for 30 s, and 72°C for 1 min. The sequences of all primers are listed in Supplementary Table 5. To ensure the credibility of the results, each sample was tested in triplicate. Using β-actin as an endogenous reference for mRNA normalization, the data were calculated using the 2^–ΔΔ*Ct*^ method and was presented as the mean ± SD using IBM SPSS statistics V22.0. Differences resulting in a *p*-value < 0.05 were considered statistically significant.

## Results

### Illumina Sequencing

To evaluate the quality of the Illumina sequencing, a total of 3,635,530,764 raw reads were generated from the 36 RNA-Seq datasets. A total of 3,455,715,304 high-quality clean reads were obtained after screening the raw reads and were used for mapping and transcriptome assembly. The average Q20, Q30, GC contents, and error rates were 96.21, 90.86, 49.52, and 0.02%, respectively, which was high enough for further analysis (Table 1).

**TABLE 1:** Raw data, clean data, quality and GC content of 36 transcriptomes from the tissues of hypothalamus-pituitary-ovarian (HPO) axis at four ages of hens.

Sample name	Raw reads	Clean reads	Q20 (Clean reads)	Q30 (Clean reads)	GC Content (clean reads)	Error rate
H15_1	107070970	103730874	94.47%	89.22%	49.01%	0.03%
H15_2	110926506	107375016	94.87%	89.02%	48.45%	0.03%
H15_3	108520724	104843110	94.60%	88.60%	49.07%	0.03%
H20_1	102591682	91978252	95.40%	88.84%	50.35%	0.02%
H20_2	93401116	89429338	97.01%	92.38%	50.04%	0.02%
H20_3	110509498	106746240	97.18%	93.01%	51.93%	0.02%
H30_1	99591894	96672562	96.45%	91.56%	48.00%	0.02%
H30_2	108078742	102427084	96.13%	90.73%	48.91%	0.02%
H30_3	112978466	110575642	96.82%	92.37%	48.24%	0.02%
H68_1	110166072	106727232	97.47%	93.72%	50.05%	0.01%
H68_2	90082030	86223358	97.03%	92.45%	49.94%	0.02%
H68_3	87295316	83762850	96.87%	92.12%	48.79%	0.02%
P15_1	102387630	98132488	96.81%	92.06%	46.33%	0.02%
P15_2	91929454	87835908	96.88%	92.17%	47.09%	0.02%
P15_3	102458208	97687892	96.92%	92.19%	48.88%	0.02%
P20_1	90529502	86091934	96.92%	92.24%	48.17%	0.02%
P20_2	92065624	88161182	97.06%	92.57%	46.17%	0.02%
P20_3	82165648	79923374	96.96%	92.40%	50.17%	0.02%
P30_1	94008258	89616974	96.92%	92.33%	50.56%	0.02%
P30_2	86961510	80863012	94.58%	87.05%	46.55%	0.03%
P30_3	87202180	80953636	94.31%	86.50%	48.45%	0.03%
P68_1	111188934	105174500	95.02%	88.63%	48.79%	0.02%
P68_2	110463626	106352586	97.09%	93.53%	44.79%	0.01%
P68_3	101323228	97473420	97.10%	93.55%	44.68%	0.01%
O15_1	93111000	86789686	96.15%	90.54%	49.53%	0.02%
O15_2	93903244	88062558	95.92%	90.01%	50.09%	0.02%
O15_3	83885806	78689920	95.97%	90.19%	49.53%	0.02%
O20_1	98291872	92073930	95.84%	89.91%	53.08%	0.02%
O20_2	89376294	83980312	95.87%	89.95%	51.09%	0.02%
O20_3	102757886	94330090	95.60%	89.30%	52.32%	0.02%
O30_1	95300758	88280870	95.36%	88.81%	52.24%	0.02%
O30_2	99835164	96702800	96.54%	91.44%	52.70%	0.02%
O30_3	143326170	131267236	95.36%	89.29%	54.16%	0.02%
O68_1	91523808	85509102	95.30%	88.72%	51.79%	0.02%
O68_2	131756526	127234318	97.37%	93.42%	51.42%	0.01%
O68_3	118565418	114036018	97.58%	93.88%	51.50%	0.01%
Total	3635530764	3455715304				
Average			96.21%	90.96%	49.52%	0.02%

*The sample name consisted of the letters and numerals. The capital letter correspond to the tissue (H: hypothalamus; P: pituitary; and O: ovary), while the numerals (15, 20, 30, and 68) correspond to the four different weeks of age and the numerals (1–3) correspond to the three samples in each age. This naming convention is the same as the following figures.*

## DEGs Analysis

**FIGURE 1:**
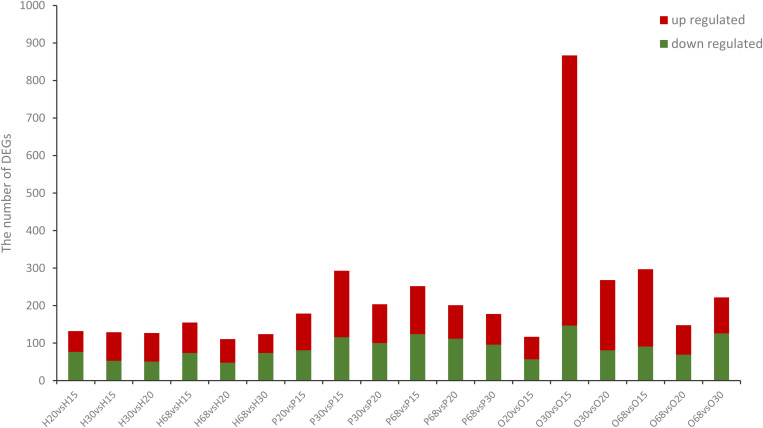
Number of DEGs in the three tissues from the HPO axis at four different ages. The name of the abscissa consists of letters and numerals, the capital letters correspond to the tissue (H: hypothalamus; P: pituitary; and O: ovary), while the numerals indicates the weeks of age (i.e., 15, 20, 30, and 68 w). This naming convention is the same in the following figures. The blue bars represent the number of significantly down-regulated DEGs while red bars represent significantly up-regulated DEGs.

**FIGURE 2:**
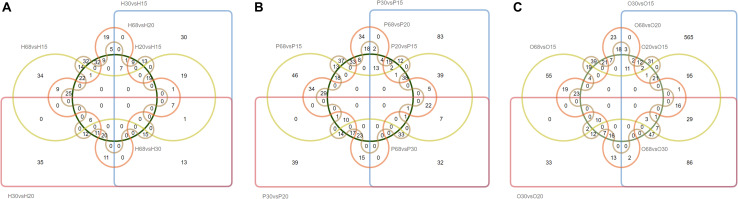
Venn diagrams of the DEGs in the three tissues of HPO axis at four ages. The capital letters in the upper left correspond to the analysis of different tissues [**(A)** hypothalamus, **(B)** pituitary, and **(C)** ovary]. This naming convention is the same as the following figures.

To investigate the key genes of the HPO axis that were involved in follicular development and ovarian function, the differentially expressed genes (DEGs) of the hypothalamus, pituitary, and ovary at the four stages of development of laying hens were analyzed (Figures 1, 2). Among the six combinations of different ages, 381 differentially expressed genes were found in the hypothalamus, of which 250 were upregulated and 230 were downregulated. A total of 622 DEGs were identified in the pituitary, including 396 upregulated and 374 downregulated genes. Moreover, in the ovary, there were 1090 DEGs, which was higher than the other tissues of the HPO axis, including 913 upregulated and 370 downregulated genes. In particular, the greatest number of DEGs (867) was obtained in the combination of O30 and O15, which reflected the initial and most active periods of the ovary Furthermore, there were 124, 178, and 222 DEGs between the 30 and 68 w periods in the hypothalamus, pituitary and ovary, respectively, which showed the changes between the peak and late laying period (Figures 1A–C). In the Venn diagrams, there were no common DEGs when considering all six combinations in each tissue. However, a total of 19, 41 and 16 common DEGs were obtained, when comparing the 20 vs 15 w, 30 vs 15 w, and 68 vs 15 w in the hypothalamus, pituitary and ovary, respectively, which corresponded with the transformation into the laying period (Figures 2A–C).

## Hierarchical Cluster Analysis

**FIGURE 3:**
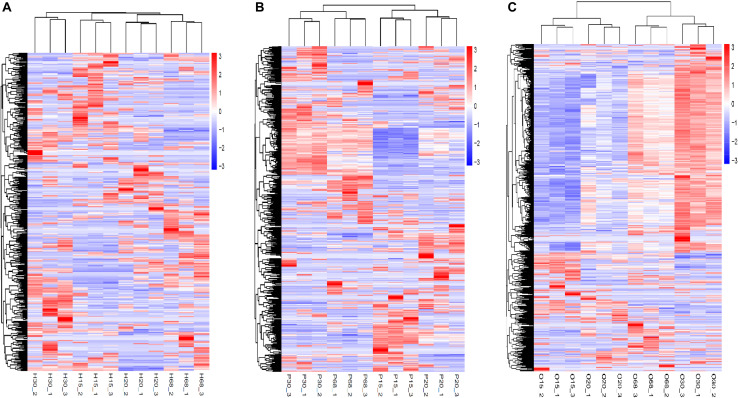
Hierarchical clustering heatmap of DEGs in the three tissues of HPO axis at four ages [**(A)** hypothalamus, **(B)** pituitary, and **(C)** ovary]. The horizontal axis below showed sample name, while the horizontal axis above showed sample clustering, the left vertical axis represented gene clustering. The color from red to blue indicates gene expression from high to low.

Hierarchical clustering heatmaps of all the DEGs with different combinations of ages in the three tissues of the HPO axis were produced (Figures 3A–C). The three samples of each age were distributed into the same sub-cluster, which indicated positive biological replicates. The DEG expression models in the pituitary and ovary were similar between 30 and 68 w, in contrast to the models at 15 and 20 w (Figures 3B,C), which may explain the variation in the ovarian development and ovulation cycles of hens.

### GO and KEGG Analysis

**FIGURE 4:**
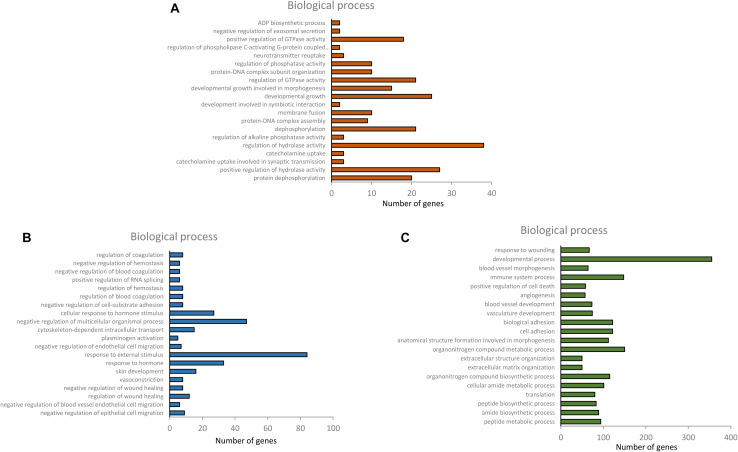
Top 20 BP terms of gene ontology of differentially expressed genes in hypothalamus, pituitary and ovary [**(A)** hypothalamus, **(B)** pituitary, and **(C)** ovary].

Upregulated and downregulated DEGs in the six combinations of each tissue were used for GO and KEGG analysis. In the GO enrichment analysis, there were 112 significantly enriched biological process (BP) items in the hypothalamus, and 178 and 709 significant BP terms were obtained in the pituitary and ovary, respectively (*p* < 0.01). Only the top 20 significant BP terms from the large dataset of each tissue are presented in Figures 4A–C. Significant BP terms in the hypothalamus were mainly involved in cell development and the regulatory processs of hormone secretion, such as development, growth, protein dephosphorylation, catecholamine uptake, protein-DNA complex assembly, regulation of GTPase activity, neurotransmitter reuptake, regulation of phospholipase C-activating G-protein coupled receptor signaling pathway, and negative regulation of exosomal secretion, which are associated with physiological processes during the different developmental stages of chickens. In the pituitary, tissue development and response to hormone stimulus were found in the main BP-enriched category, including the negative regulation of epithelial cell migration, skin development, cytoskeleton-dependent intracellular transport, cellular response to hormone stimulus, and response to hormones, which might indicate the function of the HPO axis in the regulation and developmental stages. Furthermore, a large number of significant BP terms in the ovary were obtained, which were highly relevant to follicular growth and ovulatory processes, including the cell adhesion, blood vessel development and angiogenesis, cellular amide metabolic process, multicellular organismal metabolic processes, cell differentiation, cellular response to cytokine stimulus, and response to external biotic stimuli. The concrete profiles of the GO top 20 BP terms are listed in Supplementary Tables 1–3.

**FIGURE 5:**
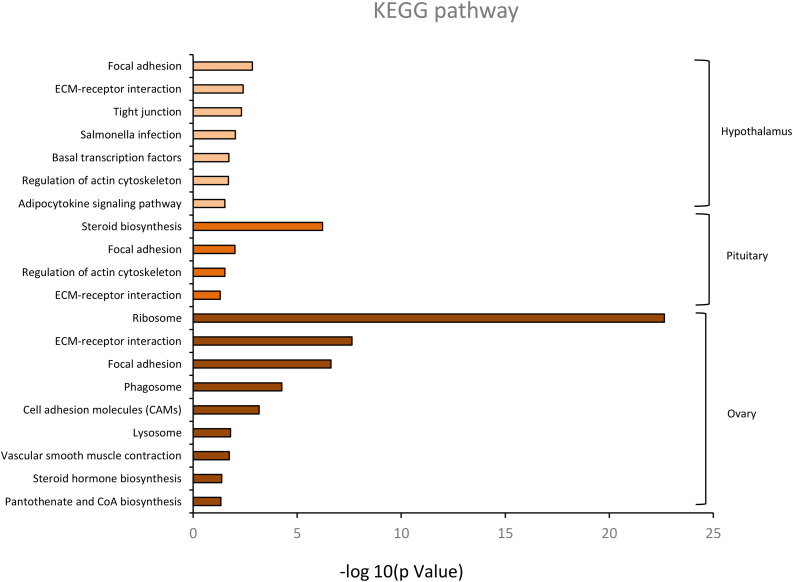
The significant KEGG classification of differentially expressed genes in hypothalamus, pituitary and ovary (*p* < 0.05).

KEGG pathway analysis of the HPO axis tissues is presented in Figure 5. It revealed that there were 113 DEGs in the hypothalamus, 193 DEGs in the pituitary, and 401 DEGs in the ovary annotated to 83, 116, and 129 KEGG pathways, respectively. Considering the different combinations of the four age groups, 32 DEGs in the hypothalamus and pituitary and 163 DEGs in the ovary were significantly enriched in 7, 4, and 9 KEGG pathways, respectively (*p* < 0.05). In these significant pathways, both focal adhesion and extracellular matrix (ECM)-receptor interaction were found in all the tissues, which were involved in cell adhesion, migration, proliferation, and differentiation, thereby mediating tissue development (Schwartz et al., 1995). In addition, five genes co-existed in the above two pathways, *COL1A1, COL4A2, PIKFYVE, PPPIR12A*, and *VWF*. Moreover, basal transcription factors and the adipocytokine signaling pathway are involved in the regulation of hypothalamic hormone secretion. The pathway of steroid biosynthesis in the pituitary is mainly involved in steroid production, which regulates many physiological processes, such as the stress response, ovarian cycle, and endocrine system (Porcu et al., 2016). However, ribosome, vascular smooth muscle contraction, steroid hormone biosynthesis, and pantothenate and CoA biosynthesis in the enriched pathways of the ovary were strongly associated with ovarian follicle development and sex hormone secretion. In particular, steroid hormone biosynthesis was primarily regulated by the HPO axis. Overall, the results of the GO and KEGG analyses highlighted tissue development with age-related alterations, and the main role of the HPO axis was in maintaining ovarian function and the ovulatory processes of the hens through the positive and negative feedback of reproductive hormones. The concrete profiles of significant KEGG pathways are listed in Supplementary Table 4.

### PPI Network

**FIGURE 6:**
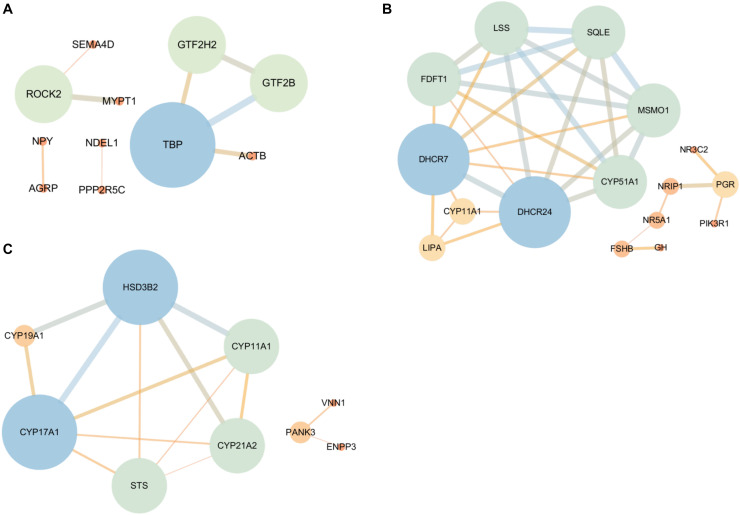
The protein-protein interaction (PPI) network of the DEGs that are associated with HPO axis function according to the GO and KEGG analyses [**(A)** Hypothalamic, **(B)** Pituitary, and **(C)** Ovary]. The bigger the node circle and the thicker the edges, the greater the connectivity among genes. The color gradient from blue to orange also corresponds with the connectivity among genes, indicating strong to weak, respectively.

To identify the candidate DEGs that were probably related to the regulation of reproductive hormone synthesis and secretion according to the top 20 enriched BP terms and significant KEGG pathways, PPI network analysis was conducted, and 43, 42, and 11 DEGs in the hypothalamus, pituitary, and ovary, respectively, were identified. In this network, only *ROCK2, TBP, GTF2H2*, and *GTF2B* were highly correlated in the hypothalamus, and are involved in the regulation of GTPase activity. In the pituitary, there were seven highly correlated genes/proteins enriched in steroid synthesis (*DHCR24, DHCR7, FDFT1, LSS, SQLE, MSMO1*, and *CYP51A1*) as well as the progesterone receptor (*PGR*) related to other proteins (*NR5A1, FSHB*, and *GH*) involved in the response to hormone stimulus from the hypothalamus and ovary. Furthermore, five hub genes (*CYP19A1, CYP17A1, CYP11A1, CYP21A2*, and *HSD3B2*) were observed in the ovary, which are crucial genes for the synthesis of estrogen, androgen, and progesterone. Moreover, pantothenate kinase 3 (*PANK3)* was found, which is involved in lipid synthesis and cholesterol, and is a necessary precursor of steroid hormone synthesis, which indicates that *PANK3* may affect the expression of sex hormones (Figure 6).

### Verification of Transcriptome Profiles

**FIGURE 7:**
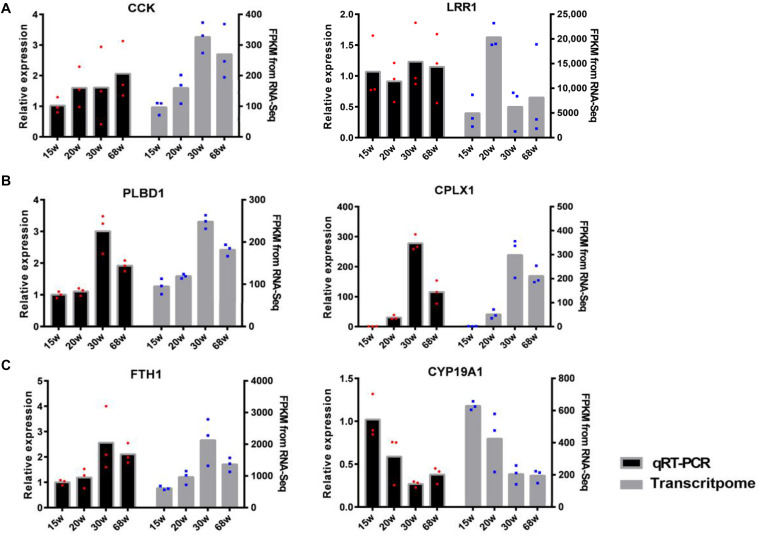
The validation of differentially expressed mRNAs between qRT-PCR and transcriptome analysis [**(A)** hypothalamic, **(B)** pituitary, and **(C)** ovary]. The left hand *y*-axis shows the value of relative expression, the right hand *y*-axis shows the value of FPKM. The dots with red and blue correspond to the three biological replicates.

Six genes with high expression from the tissues of the HPO axis were selected to validate the accuracy of the sequencing results: *CCK, LRR1, PLBD1, CPX1, FTH1*, and *CYP19A1*, with the first two genes from the hypothalamus, the second two from the pituitary, and the last two from the ovary. The consistency of these gene expression results between the transcriptome analysis and qRT-PCR was assessed (Figure 7). This proved the reliability of the transcriptome sequencing and provided confidence for further studies on the regulation of the HPO axis of follicular development and ovarian function.

## Discussion

The present study identified 381, 622, and 1090 differentially expressed genes from the hypothalamus, pituitary, and ovary in multiple comparisons among the four developmental stages of hens. A larger number of DEGs were found in the ovary compared to the other tissues, and most of them were upregulated at 30 and 68 w and were downregulated at 15 and 20 w, indicating that these genes may be associated with ovarian function and ovulation process. Some researchers have reported that the ovary of hens grows slowly before sexual maturation (18–20 weeks post hatch), and then rapidly develops until reaching the largest size with numerous ovarian follicles during the peak period of egg production (28–32 w post hatch). This could explain why the greatest number of DEGs was found in the comparison between the15 and 30 w (Figures 1–3; Waddington and Walker, 1988; Johnson, 2000; Robinson et al., 2001). Moreover, a high rate of follicular atresia occurred after the peak laying period, especially, in the late laying period and 222 DEGs were identified in ovary between the 30 and 68 w, which might be indicative of the related regulatory mechanisms (Figure 1). However, most sex-hormone genes (like GnRH1/2 and LHB) did not have the different expressions among the combination of the four ages, except for FSHB. The main reason was that exceptionally low gene expression levels were measured in the transcriptome analysis. Although many researchers have reported a significant difference in the serum sex hormones of hens at different ages, it indicated the strong action of the post-transcriptional regulation. Thus, in this study, we focused on the transcriptional regulation at the HPO axis during the different developmental stages of hens.

The significantly enriched BP terms from the GO analysis in the three tissues of the HPO axis elucidated that most of the DEGs were involved in tissue development and the regulation of hormone secretion. The BP terms of catecholamine uptake, regulation of GTPase activity, regulation of phospholipase activity, neurotransmitter reuptake, and regulation of the phospholipase C-activating G-protein coupled receptor (GPCR) signaling pathway in the hypothalamus are well known to be involved in the release and function of GnRH/GnIH, which indicated that GnRH/GnIH conducted its effects via the related biology process and stimulated/inhibited the secretion of FSH and LH in the pituitary gland and ultimately affecting the reproductive system (MacColl et al., 2002; Bédécarrats et al., 2009; Flanagan and Manilall, 2017). In the pituitary, the cellular response to hormone stimuli and the response to hormones were enriched in the top 20 significant BP terms, which reflected the function of the HPO axis. Many studies have shown that the receptors of LH and FSH widespread in ovarian follicle cells in hens, which induces the release of sex hormones (estrogen, androgen, and progesterone) in follicular theca and granulosa cells (Woods and Johnson, 2005; Johnson, 2015). Furthermore, there were an additional 709 significant BP terms identified in the ovary when compared to the other tissues, and they were mainly involved in follicular development, maturation, and ovulatory processes (Figure 4 and Supplementary Table 3). In particular, 4 of the top 20 significant BP terms of the ovary were directly related to blood vessel formation, which is an essential indicator of the selection of pre-hierarchal follicles into preovulatory follicles (Pertea et al., 2016). During follicle maturation, the production of estrogen and testosterone is primarily mediated by FSH with high expression in pre-hierarchal follicles, which then decreases in preovulatory follicles, while the secretion of progesterone surges in sensitivity to LH and the highest level occurs in the F1 follicles, which is closely linked to the regulation of the HPO axis in order to maintain the ovulatory cycle (Onagbesan and Peddie, 1989; Kato et al., 1995; Onagbesan et al., 2006).

In the analysis of the KEGG pathway, 7, 4, and 9 significantly enriched pathways were obtained in the hypothalamic, pituitary, and ovary, respectively (*p* < 0.05) (Figure 5). Focal adhesion and extracellular matrix (ECM)-receptor interaction were found to coexist in the three tissues with five co-expressed genes (*COL1A1, COL4A2, PIKFYVE, PPPIR12A*, and *VWF*), which reflected age-related alterations in tissue development (Peebles et al., 2006). Furthermore, these enriched pathways were related to the regulation and biosynthesis of reproductive hormones that are also found in the three tissues, such as basal transcription factors, adipocytokine signaling pathways, steroid biosynthesis, and steroid hormone biosynthesis. This indicates that the production of these hormones is clearly shaped by various ages (Braw-Tal et al., 2004). In addition, the enriched pathway of vascular smooth muscle contraction was found to be enriched in the ovary, which participates in the process of follicular maturation during the ovulatory cycle. These results suggest that the DEGs in the ovary are mainly involved in ovarian follicular growth, maturation, and sex hormone secretion, which is consistent with the significant BP terms in the GO enrichment analysis.

A total of 36 genes strongly associated with the regulation of hormone synthesis and secretion were observed in the PPI network analysis of the significantly enriched GO and KEGG pathways (Figures 3, 4), of which four hub genes (*ROCK2, TBP, GTF2H2*, and *GTF2B*) were found in the hypothalamus. Some researchers have reported that Rho-Kinase2 (*ROCK2*) is a key downstream target of the small GTPase Rho family, which participates in various cellular activities, such as myosin phosphorylated, actomyosin contraction, and cell motility (Godoy et al., 2011). Rahanmin-Ben Navi et al. proved that RhoA/ROCK is involved in GnRH-induced bleb formation, which might impact GnRH-stimulated gonadotropin in the pituitary via the link between signals and blebs (Rahamim-Ben Navi et al., 2017). Furthermore, TATA binding protein (*TBP*), general transcription factor IIH subunit 2 (*GTF2H2*), and general transcription factor IIB (*GTF2B*) as general transcription factors, assemble on the promoters of the gene transcribed by RNA polymerase II, which trigger transcriptional regulation by interacting with gene regulatory factors (Sperling, 2007; Bieniossek et al., 2013). In general, *TBP* interacts with TBP-associated factors (*TAFs*) to identify and activate gene transcription, of which selective promoter factor 1 (*SP-1*), as one of the TAFs, could induce GnRH responsiveness to the LHβ promoter by mediating the actions of both adenylate cyclase (AC) and protein kinase C (PKC) (Zeng et al., 2005). Moreover, *TAF9* and *GTF2H2* have been shown to play roles in the estrogen signaling pathway (Markaverich et al., 2011). This demonstrated that general transcription factors might also be involved in the complex regulation of reproductive hormones.

In the pituitary, there are seven highly related genes involved in intracellular cholesterol biosynthesis; in particular, 24-dehydrocholesterol reductase (*DHCR24)* has been shown to mediate the protective effect of IGF-1 and estrogen stimulation in the nervous system (Peri et al., 2009). Considering all of the above-related genes of cholesterol biosynthesis, a potential relationship might exist between cell cholesterol and estrogen, which may indicate a negative response to the HPO axis. Furthermore, nuclear receptor interacting protein (*NRIP1*), a co-regulator of estrogen receptor-α (ERα), directly mediates the expression of *PGR* and *NR5A1* (also called steroidogenic factor 1; *SF-1*), which are well known to respond to plasma progesterone levels and regulate reproductive development and the secretion of gonadotropin in the pituitary (Nautiyal et al., 2013). In the ovary, six genes were observed in the PPI network, of which five hub genes (*CYP19A1, CYP17A1, CYP11A1, CYP21A2*, and *HSD3B2*) participated in the biosynthesis of steroid hormones (Miller and Auchus, 2011). The remaining one- *PANK3* has been reported to mediate lipid metabolism by stimulating CoA synthesis (Dansie et al., 2014), while there was no evidence of its function in the regulation of steroid hormones. Hence, our study suggests that these novel genes may also play important roles in the function of the HPO axis.

Overall, ovarian follicle development, maturation, and its functions were mainly controlled by the HPO axis and age-related alterations, and these novel potential genes derived from the significantly enriched BP terms of the GO and KEGG pathways associated with the regulation of reproductive hormone secretion and biosynthesis might be used to further study the regulatory mechanisms of the HPO axis on the reproductive system.

In conclusion, our study presented RNA-seq analysis of the HPO axis at four ages of hens and revealed that the HPO axis could be highly associated with the regulatory mechanisms of follicular development and ovarian function through the control of sex hormone biosynthesis and secretion in the ovaries of hens. It not only provides novel candidate DEGs and potential pathways for further research, but also provides a better understanding of the HPO axis function in the reproduction system and ovulatory cycle of poultry.

## Data Availability Statement

The RNA-seq datasets generated and/or analyzed during the present study are available at NCBI project PRJNA634976 with accession number SAMN15014306–SAMN15014341.

## Ethics Statement

The animal study was reviewed and approved by the Administration of affairs concerning experimental animals (revised edition 2017) and Henan Agricultural University Institutional Animal Care and Use Committee (Permit Number: 19-0068).

## Author Contributions

YT and XK acquired the funding for the study. JL and CL performed research, analyzed the data, and wrote the manuscript. QL and GL analyzed the data and was involved in the study design. HL and WL performed the statistical analysis. YT and XK were involved in the design of the study. YT conceived the study and involved in its design and coordination. All the authors read and approved the final manuscript.

## Conflict of Interest

The authors declare that the research was conducted in the absence of any commercial or financial relationships that could be construed as a potential conflict of interest.

## Supplementary Material

Supplementary Table 1 The list of top 20 significantly enriched BP terms of all the DEGs in hypothalamus (*p* < 0.01).

Supplementary Table 2 The list of top 20 significantly enriched BP terms of all the DEGs in pituitary.

Supplementary Table 3 The list of top 20 significantly enriched BP terms of all the DEGs in ovary.

Supplementary Table 4 The list of the significant KEGG classification of differentially expressed genes.

Supplementary Table 5 The list of gene primers sequencing used for qRT-PCR validation.
