# Supplementary material for: Novel Regulatory Factors in the Hypothalamic-Pituitary-Ovarian Axis of Hens at Four Developmental Stages
Source: Front Genet. 2020 Nov 4;11:591672. doi: 10.3389/fgene.2020.591672 (PMC7672196; doi:10.3389/fgene.2020.591672)
Supplement: Supplementary Table 1 — The list of top 20 significantly enriched BP terms of all the DEGs in hypothalamus (p < 0.01). [file Table_1.DOCX]

Table S1. The list of top 20 significantly enriched BP terms of all the DEGs in hypothalamus (P<0.01)

| GO accession | Description | Term_type | P Value | DEG number | Gene name |
| --- | --- | --- | --- | --- | --- |
| GO:0006470 | protein dephosphorylation | Biological process | 9.75E-05 | 20 | FBXW11,SEMA4D,PTPN4,MARF1,SPRED1,PPP1R12A,MTMR1,NPNT,MEF2C,PTPRS,CTTNBP2NL,ENSGALG00000017031,ROCK2,PPP2R5C,WNK1,RPAP2,RNGTT,PPP4R1PTPDC1,BTRC |
| GO:0051345 | positive regulation of hydrolase activity | Biological process | 0.000174 | 27 | PSAP,MAPRE2,RAP1GAP1,LGMN,DOCK4,BLNK,ROCK2,GARNL3,DNAJB2,ENSGALG00000044260,ARHGAP29,EVI5,SNX13,NDEL1,ARHGAP21,NPNT,MYO9B,PREX1,MEF2C,ROBO1,ARHGAP44,ITGA6,TBC1D30,TBC1D4,SEMA4D,PPP1R12A,DENND1A |
| GO:0051934 | catecholamine uptake involved in synaptic transmission | Biological process | 0.000282 | 3 | PARK7,ACTB,PRKN |
| GO:0090493 | catecholamine uptake | Biological process | 0.000282 | 3 | PARK7,ACTB,PRKN |
| GO:0051336 | regulation of hydrolase activity | Biological process | 0.000391 | 38 | TBC1D4,SEMA4D,TBC1D30,DENND1A,PPP1R12A,ITGA6,ROBO1,FNIP1,MYO9B,BICD1,NDEL1,NPNT,ENSGALG00000044260,EVI5,ARHGAP29,GARNL3,RASIP1,ROCK2,MAPRE2,PSAP,MARF1,SPRED1,ARHGAP44,MEF2C,ARHGAP21,SNX13,PREX1,SBF1,DNAJB2,PPP2R5C,WNK1,BLNK,DOCK4,PARK7,PPP4R1,LGMN,RAP1GAP1,TNNT3 |
| GO:0010692 | regulation of alkaline phosphatase activity | Biological process | 0.000499 | 3 | NPNT,SEMA4D,MEF2C |
| GO:0016311 | dephosphorylation | Biological process | 0.000567 | 21 | WNK1,PPP2R5C,ROCK2,ENSGALG00000017031,BTRC,PTPDC1,RNGTT,PPP4R1,RPAP2,PPP1R12A,MARF1,SPRED1,PTPN4,SEMA4D,FBXW11,CTTNBP2NL,PSPH,MEF2C,PTPRS,NPNT,MTMR1 |
| GO:0065004 | protein-DNA complex assembly | Biological process | 0.000785 | 9 | ENSGALG00000002439,CABIN1,NAP1L4,GTF2B,SENP6,TAF7L,SHPRH,TBP,WDR18 |
| GO:0061025 | membrane fusion | Biological process | 0.000865 | 10 | UBXN2B,SNX14,ANKRD27,VPS11,PRKN,RAB7A,MFF,ANXA2,MX1,SPG11 |
| GO:0044111 | development involved in symbiotic interaction | Biological process | 0.000944 | 2 | ANXA2,NEDD4 |
| GO:0048589 | developmental growth | Biological process | 0.001069 | 25 | ARID2,MACF1,RBPJ,MEF2C,RARB,PTPRS,PLS1,NOV,S1PR1,NDEL1,NEDD4L,SPG11,SEMA4D,RARG,SLC4A10,ANXA2,MAPT,PRKN,LGMN,GH,PSAP,VCL,POU1F1,SEMA5A,NRP2 |
| GO:0060560 | developmental growth involved in morphogenesis | Biological process | 0.001071 | 15 | NEDD4L,VCL,NDEL1,S1PR1,RARB,PTPRS,MACF1,PRKN,MAPT,ANXA2,NRP2,RARG,SEMA4D,SEMA5A,SPG11 |
| GO:0043087 | regulation of GTPase activity | Biological process | 0.001129 | 21 | PREX1,MYO9B,SNX13,NDEL1,ARHGAP21,DENND1A,TBC1D4,SEMA4D,TBC1D30,ARHGAP44,ITGA6,RAP1GAP1,MAPRE2,ARHGAP29,WNK1,EVI5,ENSGALG00000044260,SBF1,GARNL3,RASIP1,DOCK4 |
| GO:0071824 | protein-DNA complex subunit organization | Biological process | 0.00124 | 10 | CABIN1,ARID2,ENSGALG00000002439,SHPRH,TBP,WDR18,TAF7L,NAP1L4,SENP6,GTF2B |
| GO:0010921 | regulation of phosphatase activity | Biological process | 0.001241 | 10 | NPNT,MEF2C,PPP4R1,ROCK2,PPP2R5C,MARF1,SPRED1,PPP1R12A,WNK1,SEMA4D |
| GO:0098810 | neurotransmitter reuptake | Biological process | 0.001267 | 3 | PARK1,ACTB,PRKN |
| GO:1900736 | regulation of phospholipase C-activating G-protein coupled receptor signaling pathway | Biological process | 0.001445 | 2 | BICD1,GRP |
| GO:0043547 | positive regulation of GTPase activity | Biological process | 0.001453 | 18 | ARHGAP29,EVI5,ENSGALG00000044260,DOCK4,GARNL3,RAP1GAP1,MAPRE2,DENND1A,TBC1D30,TBC1D4,SEMA4D,ARHGAP44,ITGA6,PREX1,NDEL1,ARHGAP21,SNX13,MYO9B |
| GO:1903542 | negative regulation of exosomal secretion | Biological process | 0.001482 | 2 | PRKN,RAB7A |
| GO:0006172 | ADP biosynthetic process | Biological process | 0.001524 | 2 | AK2,AK1 |
