# Supplementary material for: Novel Regulatory Factors in the Hypothalamic-Pituitary-Ovarian Axis of Hens at Four Developmental Stages
Source: Front Genet. 2020 Nov 4;11:591672. doi: 10.3389/fgene.2020.591672 (PMC7672196; doi:10.3389/fgene.2020.591672)
Supplement: Supplementary Table 2 — The list of top 20 significantly enriched BP terms of all the DEGs in pituitary. [file Table_2.DOCX]

Table S2. The list of top 20 significantly enriched BP terms of all the DEGs in pituitary (P<0.01)

| GO accession | Description | Term_type | P Value | DEG number | Gene name |
| --- | --- | --- | --- | --- | --- |
| GO:0010633 | negative regulation of epithelial cell migration | Biological process | 5.36E-05 | 9 | CORO1C,PDCD10,angiopoietin-2,SEMA3A,SPRED1,HMGB1,DAB2IP,MEF2C,RHOA |
| GO:0043537 | negative regulation of blood vessel endothelial cell migration | Biological process | 6.45E-05 | 6 | MEF2C,angiopoietin-2,SPRED1,RHOA,PDCD10,HMGB1 |
| GO:0061041 | regulation of wound healing | Biological process | 7.89E-05 | 12 | ANXA5,TFPI,CLASP1,FGA,ANXA2,NFE2L2,PHLDB2,FGB,CAV1,HMGB1,MYLK,FGG |
| GO:0061045 | negative regulation of wound healing | Biological process | 9.09E-05 | 8 | ANXA5,CLASP1,TFPI,ANXA2,FGA,FGG,FGB,PHLDB2 |
| GO:0042310 | vasoconstriction | Biological process | 9.75E-05 | 8 | RHOA,FGA,GRIP2,FGG,ACTA2,CAV1,FGB,HTR1B |
| GO:0043588 | skin development | Biological process | 0.00011 | 16 | COL1A2,ARRDC3,ST14,COL1A1,MAP2K1,RBPJ,STMN1,GAL,SHH,IFT74,IRF6,DHCR24,FST,PSAP,ROCK2,ABCA12 |
| GO:0009725 | response to hormone | Biological process | 0.000154 | 33 | CYP11A1,GPLD1,FSHB,BCAS3,NR6A1,MEF2C,CITED4,GKAP1,GRB14,SOCS2,TYRP1,ANXA5,NDEL1,PIK3R1,HMGB1,PGR,PRLH,RARG,SESN3,POU4F1,STXBP3,GH,NRIP1,NR3C2,EPHA5,ROBO2,TFPI,LPIN1,PKM,CAV1,NR5A1,ABCA2,ROCK2 |
| GO:0009605 | response to external stimulus | Biological process | 0.000186 | 84 | USP33,SNX14,MAPK8IP3,FGB,EIF2AK4,TFPI,CLASP1,ARF1,ANXA2,DAB2IP,FKBP51,VDAC1,LAMP2,PIKFYVE,GNG12,TMEM150C,SEMA3A,NTN1,COL1A1,RBPJ,RAB7A,SESN3,CATHL1,B3GNT2,VPS13D,GH,MX1,PRLH,PDE4D,ENSGALG00000040371,FGG,HMGB1,ENSGALG00000044478,ULK1,CACNB4,EIF4G1,TRAF3,angiopoietin-2,SHH,STMN1,DRGX,RRAGB,NRCAM,GAPDH,FGA,RHOA,CAV1,HMCN1,ENSGALG00000001912,MPP1,AOAH,POSTN,RGR,ABCC9,PIK3C3,ATF3,PUM1,STX17,PHLDB2,ROBO2,MTMR3,MAP2K1,EPHA5,LPAR1,WRN,CADPS2,TF,MYCBP2,GRP,NRXN1,RYK,NFE2L2,ANXA5,PER3,LITAF,MEF2C,CHID1,RB1,BCAS3,ACTA2,LHFPL5,AICDA,PLEKHG5,NEO1 |
| GO:0010596 | negative regulation of endothelial cell migration | Biological process | 0.000225 | 7 | HMGB1,PDCD10,angiopoietin-2,MEF2C,DAB2IP,RHOA,SPRED1 |
| GO:0031639 | plasminogen activation | Biological process | 0.000262 | 5 | FGG,FGB,DHCR24,ANXA2,FGA, |
| GO:0030705 | cytoskeleton-dependent intracellular transport | Biological process | 0.0003 | 15 | IFT74,SPAST,HOOK3,KIF13A,TRAK2,ENSGALG00000046580,RHOT1,CCDC88A,COPG2,BORCS5,SUN1,NDEL1,KIF1A,PAFAH1B1,CCDC88C |
| GO:0051241 | negative regulation of multicellular organismal process | Biological process | 0.000353 | 47 | MEF2C,CHID1,SOCS2,HOOK3,RAP1GAP2,angiopoietin-2,SHH,GAL,NPPC,PRTG,PDCD10,ZFPM1,PDE4D,RARG,RYK,NFE2L2,PIK3R1,FGG,HMGB1,ANXA5,BHLHE23,HES5,PTGER3,HPGDS,ANXA2,DAB2IP,ARRDC3,ZBTB16,WWC2,SEMA3A,NTN1,RBPJ,LAPTM4B,LPAR1,SPRED1,FSTL4,FGA,RHOA,ROCK2,HDAC7,NR5A1,FGB,CYP51A1,FRZB,CORO1C,TFPI,NF1 |
| GO:0032870 | cellular response to hormone stimulus | Biological process | 0.000359 | 27 | GH,POU4F1,SESN3,EPHA5,FSHB,NR3C2,CYP11A1,GPLD1,NRIP1,ROBO2,BCAS3,GKAP1,GRB14,SOCS2,NR6A1,MEF2C,TFPI,LPIN1,PKM,ANXA5,NDEL1,PIK3R1,PGR,NR5A1,CAV1,RARG,ROCK2 |
| GO:0010812 | negative regulation of cell-substrate adhesion | Biological process | 0.000383 | 8 | angiopoietin-2,COL1A1,NF1,CORO1C,RHOA,PIK3R1,BCAS3,PHLDB2 |
| GO:0030193 | regulation of blood coagulation | Biological process | 0.000399 | 8 | FGG,FGB,CAV1,NFE2L2,FGA,ANXA2,TFPI,ANXA5, |
| GO:1900046 | regulation of hemostasis | Biological process | 0.000399 | 8 | NFE2L2,CAV1,FGB,FGG,ANXA2,FGA,TFPI,ANXA5, |
| GO:0033120 | positive regulation of RNA splicing | Biological process | 0.000417 | 6 | PIK3R1,ZPR1,SETX,THRAP3,PRDX6,HSPA8, |
| GO:0030195 | negative regulation of blood coagulation | Biological process | 0.000511 | 6 | TFPI,FGB,FGG,ANXA2,FGA,ANXA5 |
| GO:1900047 | negative regulation of hemostasis | Biological process | 0.000511 | 6 | TFPI,FGB,FGG,FGA,ANXA2,ANXA5 |
| GO:0050818 | regulation of coagulation | Biological process | 0.000545 | 8 | TFPI,ANXA5,NFE2L2,CAV1,FGB,FGG,ANXA2,FGA |
