# Supplementary material for: Novel Regulatory Factors in the Hypothalamic-Pituitary-Ovarian Axis of Hens at Four Developmental Stages
Source: Front Genet. 2020 Nov 4;11:591672. doi: 10.3389/fgene.2020.591672 (PMC7672196; doi:10.3389/fgene.2020.591672)
Supplement: Supplementary Table 3 — The list of top 20 significantly enriched BP terms of all the DEGs in ovary. [file Table_3.DOCX]

Table S3. The list of top 20 significantly enriched BP terms of all the DEGs in ovary (P<0.01)

| GO accession | Description | Term_type | P Value | DEG number | Gene name |
| --- | --- | --- | --- | --- | --- |
| GO:0006518 | peptide metabolic process | Biological  process | 2.28E-19 | 94 | RPL11,RPL27A,MRPS16,LARS,RPS3,RPL19,EEF2,RPS15A,RPL34,EIF3G,RPL24,RPL12,UBA52,CNOT7,NFE2L2,RPL35,RNASE6,THBS1,RPL32,RPS3A,RPL13,GCLM,RPS13,RPS16,CPQ,EIF4A2,RPL15,RPS26,RPS11,RPL8,STK3,RPL14,CORIN,FOXO3,EEF1A2,RPL27,GSTA3,EIF4E3,RPL17,RPS24,DAPK1,EEF1D,RPS6,RPL29,PTGES,GPX1,MRPS9,PUM1,RPL31,RPL10A,RPL22,RPL4,GSTA2,RPS20,RPL36,RPL21,RPS12,RPL6,RPS2,RPS4X,GNB2L1,GCN1,RPL26L1,RPSAP58,RPL39,RPS14,RPL37A,MGST2,RPS15,MME,RPS7,LNPEP,RPS28,RPS29,FAM129A,RPS21,RPL5,TPR,TRHDE,RPL9,RPL35A,C7,RPS27,IARS2,RPL36AL,RPS19,RPL37,CPXM1,ENSGALG00000015617,NEMF,RPL3,RPS17,TNRC6C,CTSH |
| GO:0043604 | amide biosynthetic process | Biological  process | 2.33E-19 | 89 | RPL37A,MGST2,RPS15,SMPD4,RPS28,RPS29,RPS7,RPS21,FAM129A,RPL5,TPR,RPL35A,RPL9,C7,RPL36AL,RPS19,ENSGALG00000015617,RPL37,IARS2,RPS27,RPL3,NEMF,SGMS2,RPS17,TNRC6C,RPL10A,RPL31,PUM1,RPL4,RPL22,RPS20,RPL36,RPL21,RPS12,RPL6,RPS4X,RPS2,RPSAP58,GNB2L1,GCN1,RPL26L1,RPS14,RPL39,RPS3A,GCLM,RPL13,EIF4A2,RPL15,P2RX1,RPS13,RPS16,RPL8,STK3,RPS26,RPS11,RPL14,FOXO3,RPL27,EEF1A2,DAPK1,RPS24,EEF1D,RPL17,EIF4E3,RPL29,MRPS9,RPS6,MRPS16,RPL27A,RPL11,RPS3,LARS,RPL19,EEF2,EIF3G,RPS15A,RPL34,CNOT7,NFE2L2,ASNS,RPL24,RPL12,ASS1,UBA52,RPL35,RNASE6,THBS1,RPL32,ASAH1 |
| GO:0043043 | peptide biosynthetic process | Biological  process | 3.13E-19 | 83 | RPL6,RPS12,RPS2,RPS4X,RPL26L1,GCN1,GNB2L1,RPSAP58,RPL39,RPS14,PUM1,RPL31,RPL10A,RPL22,RPL4,RPS20,RPL21,RPL36,IARS2,RPS27,RPL37,ENSGALG00000015617,RPS19,RPL36AL,NEMF,RPL3,RPS17,TNRC6C,RPS15,MGST2,RPL37A,RPS7,RPS29,RPS28,RPL5,TPR,FAM129A,RPS21,C7,RPL9,RPL35A,RPL34,RPS15A,EIF3G,UBA52,RPL24,RPL12,NFE2L2,CNOT7,RNASE6,RPL35,RPL32,THBS1,RPL27A,RPL11,MRPS16,LARS,RPS3,RPL19,EEF2,RPL27,EEF1A2,FOXO3,RPL17,EIF4E3,RPS24,DAPK1,EEF1D,RPS6,MRPS9,RPL29,RPL13,GCLM,RPS3A,RPS16,RPS13,RPL15,EIF4A2,RPS11,RPS26,STK3,RPL8,RPL14 |
| GO:0006412 | translation | Biological | 1.72E-18 | 80 | RPL14,RPS26,RPS11,RPL8,STK3,RPS13,RPS16,EIF4A2,RPL15,RPS3A,RPL13,RPS6,RPL29,MRPS9,EIF4E3,RPL17,RPS24,DAPK1,EEF1D,FOXO3,EEF1A2,RPL27,EEF2,RPL19,LARS,RPS3,RPL11,RPL27A,MRPS16,THBS1,RPL32,RPL35,RNASE6,RPL12,RPL24,UBA52,CNOT7,RPS15A,RPL34,EIF3G,RPL9,RPL35A,C7,FAM129A,RPS21,TPR,RPL5,RPS7,RPS28,RPS29,RPS15,RPL37A,TNRC6C,RPS17,NEMF,RPL3,RPS27,IARS2,RPL36AL,RPS19,RPL37,-,RPL36,RPL21,RPS20,RPL22,RPL4,RPL31,PUM1,RPL10A,RPL39,RPS14,GCN1,GNB2L1,RPL26L1,RPSAP58,RPS2,RPS4X,RPS12,RPL6 |
| GO:0043603 | cellular amide metabolic process | process | 7.71E-18 | 101 | CORIN,RPL27,EEF1A2,FOXO3,EIF4E3,RPL17,GSTA3,DAPK1,RPS24,EEF1D,RPS6,MRPS9,GPX1,PTGES,RPL29,RPL13,GCLM,RPS3A,CPQ,RPS13,RPS16,RPL15,P2RX1,EIF4A2,RPS11,RPS26,STK3,RPL8,RPL14,RPL34,RPS15A,EIF3G,UBA52,ASS1,RPL24,ASNS,RPL12,NFE2L2,CNOT7,RNASE6,RPL35,ASAH1,THBS1,RPL32,RPL11,RPL27A,MRPS16,LARS,RPS3,RPL19,EEF2,IARS2,RPS27,SGPL1,ENSGALG00000015617,CPXM1,RPL37,RPL36AL,RPS19,NEMF,SGMS2,RPL3,RPS17,TNRC6C,CTSH,RPS15,MGST2,RPL37A,MME,RPS7,RPS29,SMPD4,LNPEP,RPS28,TPR,RPL5,RPS21,FAM129A,TRHDE,RPL9,RPL35A,C7,RPL6,RPS12,RPS2,RPS4X,GCN1,GNB2L1,RPL26L1,RPSAP58,RPL39,RPS14,RPL31,PUM1,RPL10A,RPL22,RPL4,GSTA2,RPS20,RPL21,RPL36 |
| GO:1901566 | organonitrogen compound biosynthetic process | Biological | 4.80E-15 | 115 | APOA1,EEF2,ELOVL1,PRPS2,RPL19,UROD,ACOT7,RPS3,LCAT,LARS,MRPS16,RPL27A,RPL11,THBS1,RPL32,ASAH1,RNASE6,RPL35,NFE2L2,CNOT7,UBA52,ASS1,RPL12,ASNS,RPL24,EIF3G,RPL34,RPS15A,RPL14,PALM,ADAL,STK3,RPL8,RPS11,RPS26,RPL15,P2RX1,EIF4A2,VDAC2,RPS13,RPS16,GCLM,RPL13,RPS3A,MRPS9,RPL29,RPS6,RPS24,DAPK1,PURH,EEF1D,EIF4E3,RPL17,RPL27,EEF1A2,FOXO3,HAS2,NFKB1,RPL21,RPL36,ENO1,RPS20,RPL4,RPL22,RPL10A,PUM1,GLUL,RPL31,RPS14,RPL39,CSGALNACT1,RPSAP58,RPL26L1,GCN1,GNB2L1,RPS4X,RPS2,RPL6,RPS12,ENSGALG00000008518,RPL9,C7,RPL35A,ENSGALG00000015617,PID1,RPL5,TPR,PLOD2,FAM129A,RPS21,RPS29,RPS28,SMPD4,ENSGALG00000015617,RPS7,MGST2,RPS15,RPL37A,TGFB1,TNRC6C,AK1,ADA2,RPS17,RPL3,PANK3,SGMS2,NEMF,RPL37,B3GNT9,ENSGALG00000015617,RPL36AL,RPS19,IARS2,RPS27,ATP2B4 |
| GO:0030198 | extracellular matrix organization | process | 6.69E-15 | 50 | GSN,CSGALNACT1,ITGA8,COL1A2,HSPA8,DPT,MFAP5,VWA1,COL4A2,CRISPLD2,MYH11,LCP1,NID1,PDGFRA,FAP,C1QC,SULF1,PHLDB2,MMP2,POSTN,COL8A1,VTN,IHH,AEBP1,COL5A1,RAMP2,COL5A2,COLGALT1,AGT,LUM,COL4A1,CLASP2,MMP1,RB1,HAS2,MMP9,ANXA2,PLOD2,ADAMTS2,COL12A1,COL1A1,LOXL2,CLASP1,MMP11,FBLN2,TGFB1,ELF3,FBLN1,MMP13,CD36 |
| GO:0043062 | extracellular structure organization | Biological | 9.79E-15 | 50 | COL5A1,IHH,AEBP1,COL5A2,RAMP2,POSTN,COL8A1,PHLDB2,MMP2,VTN,NID1,LCP1,PDGFRA,MYH11,SULF1,FAP,C1QC,CSGALNACT1,ITGA8,COL1A2,GSN,MFAP5,VWA1,COL4A2,DPT,CRISPLD2,HSPA8,ELF3,FBLN1,MMP13,FBLN2,MMP11,TGFB1,CD36,COL12A1,ADAMTS2,ANXA2,PLOD2,LOXL2,CLASP1,COL1A1,CLASP2,MMP1,RB1,COL4A1,HAS2,MMP9,AGT,LUM,COLGALT1 |
| GO:1901564 | organonitrogen compound metabolic process | process | 4.71E-12 | 150 | ADAL,RPS26,RPS11,EIF4A2,RPL15,RPS16,CPQ,GCLM,RPL13,RPL29,GPX1,EEF1D,RPS24,RHOQ,GSTA3,EIF4E3,RPL17,EEF1A2,LYG2,NFKB1,ITIH5,EEF2,PRPS2,ACOT7,UROD,RPS3,LARS,LCAT,MRPS16,RPL11,RPL27A,DCN,RPL32,HSPA8,ASAH1,RPL35,RNASE6,MAOA,CNOT7,NFE2L2,RPL24,RHOA,RPS15A,RPL34,C7,RPL9,RPL35A,P4HA3,RPS21,PLOD2,RPL5,RPS28,SMPD4,RPS7,ENSGALG00000037869,MME,TGFB1,RPL37A,ENSGALG00000037869,RPS17,HTR2A,ADA2,RPL3,RPL36AL,B3GNT9,RPL37,ENSGALG00000015617,ATP2B4,SGPL1,IARS2,RPS27,RPL21,ENO1,GSTA2,RPL4,RPL22,ENPP3,RPL10A,HK2,RPL31,RPS14,RPSAP58,CSGALNACT1,NDUFS1,GCN1,GDA,RPS4X,RPS2,RPL6,TPI1,PALM,RPL14,RPL8,STK3,P2RX1,RPS13,VDAC2,RPS3A,PTGES,MRPS9,RPS6,DAPK1,PURH,COMT,FOXO3,RPL27,HAS2,CORIN,APOA1,NNT,RPL19,ELOVL1,AADAT,THBS1,DPYS,ASNS,RPL12,UBA52,ASS1,EIF3G,ENSGALG00000015240,TRHDE,PID1,FAM129A,TPR,LNPEP,RPS29,MGST2,RPS15,TNRC6C,CTSH,AK1,AHCYL2,NEMF,SGMS2,PANK3,RPS19,CPXM1,RPL36,RPS20,ENSGALG00000008518,LYVE1,PUM1,GLUL,LYZ,RPL39,RPL26L1,GNB2L1,ENSGALG00000008518,RPS12 |
| GO:0048646 | anatomical structure formation involved in morphogenesis | Biological | 2.44E-11 | 112 | FOXP3,PHLDB2,COL8A1,ACTN1,TAL1,ADGRG6,PTPRB,HMGB1,RSPO3,DCN,TA3,WDR1,COL5A1,STAB1,ENSGALG00000006705,COL5A2,CTGF,RNASE6,FN1,CDH13,THBS1,RARG,C3,PDGFRA,EVI5L,LMOD1,NRP2,RHOA,ENSGALG00000029540,ENSGALG00000002012,EHD2,STK3,COL1A1,APOLD1,PECAM1,SALL4,CLASP1,ADAMTS1,SPARC,ZEB2,ITGB5,TEK,CD36,CAV1,ST14,ENSGALG00000007646,TCF21,TGM2,ADIPOR2,TIE1,GPX1,SLC40A1,JCAD,PTEN,MMP9,PLEKHO1,PLCD1,SH3PXD2A,MMP2,THY1,ITGA7,MEIS1,VTN,CDH5,UGT8,GLUL,HK2,IHH,RAMP2,GAB1,SBNO2,NRP1,PGM5,CD81,ITGA8,CEP120,PTPN6,COL6A1,MYL9,SLIT2,SPI1,SULF1,ANXA2,MAFB,COL12A1,LOXL2,TGFB1,RNASE4,ENSGALG00000007007,ITGAV,EDNRA,ENSGALG00000024379,RPS7,TPM1,DAB2IP,FHL2,MEOX2,CAPN2,ITGB2,ODF2L,RELN,C3AR1,HPSE,PIKFYVE,ACTA1,CTSH,PDCD10,COL4A1,RBPJ,ANXA1,CLASP2,BMPER |
| GO:0007155 | cell adhesion | process | 4.65E-11 | 122 | FOXP3,PHLDB2,COL6A2,CD63,ACTN1,COL8A1,ENSGALG00000031794,LGALS3,APOA1,PCDH1,HMGB1,SPON2,COL5A1,ENSGALG00000031430,STAB1,NINJ1,VCL,CTGF,GSN,DOCK8,CD83,FN1,IL15,CDH13,PTPRC,THBS1,PCDHGC3,ENSGALG00000015032,ENSGALG00000003283,B2M,UTRN,NID1,FLOT2,ASS1,RHOA,ENSGALG00000029540,TMEM131L,ENSGALG00000002012,ROBO2,COL1A1,CLASP1,PECAM1,ITGA1,FBLN2,PARVG,ITGB5,TEK,VCAN,CD36,CD44,UNC5D,CAV1,ENSGALG00000006851,GPR183,BVES,PCDH15,TGM2,RPS6,DLC1,TNC,Ii,ENSGALG00000002643,FGG,PTEN,HAS2,CNTN5,FZD4,MMP2,VWF,ENSGALG00000005257,POSTN,THY1,ITGA7,VTN,CDH5,GPNMB,LYVE1,IHH,CD93,CDH11,FNDC3A,RPL22,ENSGALG00000004960,PXN,NRP1,ADIPOQ,MXRA8,RPSAP58,CD81,ITGA8,PTPN6,LMO1,NCAM2,LCP1,FYN,PKD1,SULF1,S100A10,RAB27A,MAFB,PRKCZ,VEZT,CTNNA1,LAMA4,CSTA,TGFB1,CERCAM,ITGAV,FBLN1,LYN,ENSGALG00000052798,IRF1,ITGB2,ENSGALG00000041298,PPP1R12A,LIMS2,HPSE,JAK2,ENSGALG00000043610,ANXA1,WNK1,NKX2-3 |
| GO:0022610 | biological adhesion | Biological | 6.38E-11 | 122 | ENSGALG00000002643,FGG,PTEN,TNC,Ii,HAS2,BVES,ENSGALG00000006851,GPR183,DLC1,PCDH15,RPS6,TGM2,ITGB5,TEK,VCAN,FBLN2,ITGA1,PARVG,UNC5D,CAV1,CD44,CD36,ROBO2,TMEM131L,ENSGALG00000002012,PECAM1,CLASP1,COL1A1,B2M,UTRN,NID1,ENSGALG00000015032,ENSGALG00000003283,ENSGALG00000029540,FLOT2,ASS1,RHOA,IL15,DOCK8,CD83,FN1,CTGF,GSN,PTPRC,THBS1,CDH13,PCDHGC3,COL5A1,SPON2,VCL,ENSGALG00000031430,STAB1,NINJ1,COL8A1,ACTN1,PHLDB2,FOXP3,COL6A2,CD63,LGALS3,APOA1,PCDH1,HMGB1,ENSGALG00000031794,ANXA1,ENSGALG00000043610,JAK2,NKX2-3,WNK1,LIMS2,ITGB2,ENSGALG00000041298,PPP1R12A,HPSE,FBLN1,ITGAV,LYN,TGFB1,CERCAM,IRF1,ENSGALG00000052798,MAFB,CTNNA1,VEZT,PRKCZ,RAB27A,LAMA4,CSTA,NCAM2,LCP1,S100A10,SULF1,PKD1,FYN,RPSAP58,ITGA8,CD81,ADIPOQ,NRP1,MXRA8,LMO1,PTPN6,FNDC3A,LYVE1,IHH,CD93,CDH11,PXN,RPL22,ENSGALG00000005257,POSTN,ITGA7,THY1,CNTN5,MMP2,FZD4,VWF,ENSGALG00000005257,CDH5,GPNMB,VTN |
| GO:0001944 | vasculature development | process | 7.33E-11 | 74 | GPX1,TIE1,ADIPOR2,TCF21,BVES,ENSGALG00000007646,PLCD1,HAS2,PTEN,JCAD,PECAM1,COL1A1,APOLD1,ENSGALG00000002012,CAV1,TEK,SPARC,CDH13,NTRK2,THBS1,DNM2,RNASE6,CTGF,ENSGALG00000029540,RHOA,NRP2,PDGFRA,C3,HMGB1,PTPRB,TAL1,STAB1,COL5A1,DCN,RSPO3,CTSH,HPSE,SGCB,C3AR1,BMPER,ACTA2,ANXA1,RBPJ,COL4A1,SGPL1,PDCD10,LOXL2,LAMA4,ANXA2,MEOX2,DAB2IP,EDNRA,ENSGALG00000024379,RNASE4,ITGAV,TGFB1,COL1A2,NRP1,SULF1,PKD1,SLIT2,SPI1,AQP1,CDH5,MEIS1,THY1,MMP2,FZD4,GAB1,RAMP2,MYLK,IHH,GLUL,HK2 |
| GO:0001568 | blood vessel development | Biological  process | 7.41E-11 | 73 | LAMA4,LOXL2,ANXA2,DAB2IP,MEOX2,TGFB1,EDNRA,ENSGALG00000024379,RNASE4,ITGAV,HPSE,CTSH,C3AR1,BMPER,COL4A1,SGPL1,PDCD10,ACTA2,ANXA1,RBPJ,MEIS1,AQP1,CDH5,FZD4,MMP2,THY1,RAMP2,GAB1,IHH,GLUL,HK2,MYLK,NRP1,COL1A2,SLIT2,SPI1,SULF1,PKD1,APOLD1,COL1A1,PECAM1,ENSGALG00000002012,CAV1,SPARC,TEK,TIE1,ADIPOR2,GPX1,ENSGALG00000007646,BVES,TCF21,HAS2,PLCD1,PTEN,JCAD,PTPRB,HMGB1,TAL1,STAB1,DCN,RSPO3,COL5A1,DNM2,NTRK2,THBS1,CDH13,CTGF,RNASE6,RHOA,ENSGALG00000029540,C3,NRP2,PDGFRA |
| GO:0001525 | angiogenesis | Biological  process | 1.20E-10 | 57 | TAL1,THY1,PTPRB,MEIS1,CDH5,HMGB1,GLUL,HK2,RSPO3,DCN,IHH,STAB1,RAMP2,GAB1,NRP1,CTGF,RNASE6,THBS1,CDH13,C3,PDGFRA,NRP2,SPI1,RHOA,SLIT2,SULF1,ENSGALG00000029540,ANXA2,ENSGALG00000002012,APOLD1,PECAM1,LOXL2,SPARC,ITGAV,RNASE4,EDNRA,ENSGALG00000024379,TEK,DAB2IP,MEOX2,CAV1,ENSGALG00000007646,C3AR1,TCF21,HPSE,ADIPOR2,TIE1,CTSH,GPX1,PDCD10,COL4A1,JCAD,RBPJ,ANXA1,PTEN,BMPER,PLCD1 |
| GO:0010942 | positive regulation of cell death | Biological  process | 1.81E-10 | 58 | TLR4,TOP2A,CTSC,HMGB1,TNFAIP8,SYCE3,RASSF2,RPL11,ATF3,NACC2,ADIPOQ,GNB2L1,GSN,RAPGEF2,BID,ANXA5,THBS1,PTPRC,RARG,ENSGALG00000054198,ENSGALG00000001049,SIAH1,FAP,FNIP1,FYN,SLIT2,STK3,CTNNA1,C1QA,RARB,ENSGALG00000024379,LYN,RPS7,CD36,DAB2IP,kcnma1,P2RX1,CAV1,AGT,DAPK1,LRP1,CALHM2,TGM2,RPS6,DLC1,CTSH,INPP5D,TNFRSF10B,ANXA1,PDCD4,PTEN,NTRK3,MMP9,FRZB,TNFSF10,FOXO3,CASP18,EEF1A2 |
| GO:0002376 | immune system process | Biological  process | 2.38E-10 | 148 | ATP11C,ANXA1,RPS19,RB1,OXSR1,NKX2-3,ITGB2,C3AR1,CTSH,LYN,FGR,IRF1,DAB2IP,ANXA2,RAB27A,CCR5,LCP1,C1S,CADM1,CYBB,FLNB,FLI1,ADIPOQ,CNN2,CD81,RPL39,LMO1,PUM1,IHH,ENSGALG00000004960,C1QB,MEIS1,VTN,TIFA,SLC40A1,INPP5D,BF1,RARRES2,MMP9,ENSGALG00000040371,FOXO3,SH3PXD2A,CASP18,GPR183,DAPK1,TCF21,BVES,LY86,RPS6,PRDX1,FTH1,ENSGALG00000014585,PIK3AP1,TTBK1,RAB4A,JCHAIN,CCR2,PTN,STK3,ENSGALG00000015032,C3,CDC42EP4,FLOT2,BLB1,GSN,IL15,THBS1,PTPRC,RARG,MFAP5,CSF3R,TLR4,ACTN1,TAL1,CYP19A1,ENSGALG00000031794,ERBIN,TCIM,APOA1,ENSGALG00000022875,BLNK,SGPL1,JAK2,RBPJ,WNK1,TNFSF10,DMB2,RPS17,KMT2E,TGFB1,AVD,MAFB,PRKCZ,C1QA,C7,FYN,SPI1,SLIT2,ILF2,PTPN6,HOXB4,RPS14,ENPP3,RPL22,SBNO2,CSF1,THY1,TOP2A,GPNMB,CTSC,Ii,RPS24,IL1RL1,GPX1,ITGA1,CHGA,CD36,CAV1,ENSGALG00000006756,ACOD1,TMEM131L,IFIT5,PECAM1,PDGFRA,PGM3,B2M,C1QC,FNIP1,EML1,RHOA,BF2,CNOT7,NFE2L2,C1R,DOCK8,CD83,CIAPIN1,RASSF2,SERPINB10B,SPON2,WDR1,CFD,FOXP3,LGMN,EEF2,LGALS3,HMGB1,BLB2 |
| GO:0048514 | blood vessel morphogenesis | Biological  process | 4.13E-10 | 64 | C3AR1,HPSE,CTSH,SGPL1,COL4A1,PDCD10,ANXA1,RBPJ,BMPER,ANXA2,LOXL2,TGFB1,EDNRA,ENSGALG00000024379,RNASE4,ITGAV,DAB2IP,MEOX2,NRP1,SPI1,SLIT2,SULF1,FZD4,THY1,MEIS1,CDH5,IHH,GLUL,HK2,MYLK,RAMP2,GAB1,ENSGALG00000007646,BVES,TCF21,ADIPOR2,TIE1,GPX1,PTEN,JCAD,HAS2,PLCD1,ENSGALG00000002012,APOLD1,PECAM1,SPARC,TEK,CAV1,CTGF,RNASE6,THBS1,NTRK2,CDH13,C3,NRP2,PDGFRA,RHOA,ENSGALG00000029540,TAL1,PTPRB,HMGB1,DCN,RSPO3,STAB1 |
| GO:0032502 | developmental process | Biological process | 4.55E-10 | 355 | PTPRC,CRISPLD2,MFAP5,HTR2C,C2H8ORF22,IL15,NEFM,GSN,CTSB,FLOT2,CTBP1,CDC42EP4,CLEC3B,C3,UNC79,APOA1,BLNK,-,TLR4,BBX,OSTN,ADGRG6,PHLDB2,STAB1,IL1RAP,SUCO,TA3,DLC1,TGM2,TCF21,BVES,-,GPR183,FOXO3,PLEKHO1,SH3PXD2A,NAV2,SOCS3,ARHGAP12,SLC40A1,TPI1,DACH2,ADAMTS2,STK3,SEMA6D,ITGB5,MMP13,BDNF,ADAMTS1,PARVG,FLI1,PGM5,GNB2L1,PKD1,MYH11,MEIS1,ITGA7,MMP2,C1QB,SVIL,GAB1,ATRN,PLS1,-,IFITM3,CTSH,PIKFYVE,SGCB,ITGB2,NKX2-3,VAT1,TEKT1,CEP131,TAGLN2,FRZB,ATP11C,RB1,OLFML3,ANXA1,OGDH,PID1,CTNNA1,COL12A1,MEOX2,DAB2IP,FGR,LDLRAD4,ENSGALG00000024379,LYN,MMP11,NTRK2,LHFPL2,RNASE6,HSD17B4,FAM172A,RHOA,EML1,B2M,PDGFRA,PGM3,NID1,TRPV2,LGMN,COL6A2,RSPO3,RASSF2,SLA,DOCK7,PCDH15,ST14,RNF2,PLCD1,CLMP,JCAD,EPB41,STMN1,PTEN,Ii,HSPB7,ROBO2,MAPK8IP3,CEP162,UNC5D,CAV1,CD36,SFTPA2,TEK,VCAN,FBLN2,MEF2D,ACP5,HOXB4,CEP120,PTPN6,CSGALNACT1,NRP1,BBS9,SULF1,RPS4X,FRMD6,FYN,SPI1,SLIT2,CDH5,AQP1,GNG12,CSF1,THY1,SBNO2,SPG11,MYLK,HK2,PI15,CDH11,ACTA1,KMT2E,MARK1,WNK1,RCAN1,SGPL1,ETV3,LOXL2,SEMA3D,EHMT1,PRKCZ,TDRD1,HOPX,CAPN2,DHRS3,ITGAV,ENSGALG00000007007,MME,MAP3K4,EDNRA,TGFB1,CDH13,THBS1,RARG,DNM2,HOXC8,TMEM119,ENSGALG00000054198,MEIOC,SIAH1,USP33,NRP2,DPYSL3,EGFL7,TCIM,ACTN1,COL8A1,TAL1,CYP19A1,CD63,CSF3R,ENSGALG00000015656,RNF6,COL5A2,VCL,COL5A1,ANKLE2,TRIP12,TIE1,RPS6,ADIPOR2,COMT,CENPF,CASP18,MMP9,RARRES2,HAS2,LIPA,INPP5D,SALL4,PALM,MUSTN1,CLASP1,SINHCAF,APOLD1,COL1A1,PTN,ANKRD27,ENSGALG00000002012,SLC39A12,TRAK2,SIPA1L1,MBP,CNN2,COL1A2,ITGA8,CD81,ADIPOQ,MXRA8,RAPGEF2,FLNB,RYR2,PCK1,ZNF521,COL6A1,UGT8,VTN,SOCS2,POSTN,HOXB5,FZD4,PUM1,GLUL,SETX,IHH,PALMD,LIMS2,C3AR1,MGP,RPS19,ACTA2,CLASP2,NTRK3,PDCD10,NCS1,GDPD5,RAB27A,ANXA2,IRF1,PRRX2,FBLN1,CIAPIN1,TAGLN,HSPA8,ASAH1,CD83,FN1,CTGF,PLS3,ENSGALG00000029540,NFE2L2,RPL24,C1QC,FNIP1,EVI5L,LMOD1,TXNIP,LGALS3,HMGB1,PTPRB,EEF2,FOXP3,NINJ1,ENSGALG00000006705,NFATC1,SERPINB10B,DCN,WDR1,GPX1,MATN3,RHOQ,ENSGALG00000008297,KIAA0319,MARF1,PDCD4,TNC,PECAM1,CYP17A1,BASP1,FABP4,ACOD1,EHD2,TMEM131L,ANKRD6,GNB1,ZEB2,GCLM,FOXN3,SPARC,RPS14,EFEMP1,VWA1,S100A10,PGR,NRTN,MYL9,GLIPR2,TRPC4,TOP2A,CTSC,HTRA1,RPL22,COTL1,RAMP2,ATF3,FNDC3A,PDLIM3,STOX2,HPSE,AGT,RELN,HTR2A,ODF2L,BMPER,RBPJ,CTSK,METRNL,JAK2,COL4A1,C1QA,RARB,LAMA4,CSTA,MAFB,FHL2,EXFABP,RPS7,TPM1,RNASE4,ELF3,SEMA6B |
| GO:0009611 | response to wounding | Biological  process | 6.46E-10 | 67 | NTRK3,ANXA1,METRNL,RB1,RPS19,JAK2,PDCD10,HPSE,F8,LYN,FBLN1,TGFB1,RAB27A,ANXA2,HOPX,RARB,TFPI2,CD81,CNN2,ADIPOQ,ACP5,F13A1,PTPN6,ENPP3,MYLK,SBNO2,F5,VWF,AQP1,CDH5,FGG,PTEN,SOCS3,NFKB1,PDCD4,TNC,ENSGALG00000040371,GPX1,IL1RL1,ADIPOR2,PIK3AP1,CAV1,P2RX1,CD36,PPL,FABP4,PROS1,CLASP1,PECAM1,MUSTN1,PDGFRA,C3,DPYSL3,NFE2L2,RHOA,ANXA5,FN1,THBS1,COL5A1,WFDC1,NINJ1,CYP19A1,DST,TLR4,PHLDB2,HMGB1,APOA1 |
