# Supplementary material for: Novel Regulatory Factors in the Hypothalamic-Pituitary-Ovarian Axis of Hens at Four Developmental Stages
Source: Front Genet. 2020 Nov 4;11:591672. doi: 10.3389/fgene.2020.591672 (PMC7672196; doi:10.3389/fgene.2020.591672)
Supplement: Supplementary Table 4 — The list of the significant KEGG classification of differentially expressed genes. [file Table_4.DOCX]

TableS4. The list of the significant KEGG classification of differentially expressed genes (P<0.05)

| Tissue | Term | Pathways | P value | DEGs number | Gene name |
| --- | --- | --- | --- | --- | --- |
| Hypothala-mus | gga04510 | Focal adhesion | 0.00144056 | 13 | COL1A1,PPP1R12A,COL4A2,ACTB,VWF,  SPP1,VCL,ITGA3,ENSGALG00000042388,  FLNB,ITGA6 |
|  | gga04512 | ECM-receptor interaction | 0.00396741 | 7 | COL4A2,COL1A1,VWF,SPP1,ITGA3,  ENSGALG00000042388,ITGA6 |
|  | gga04530 | Tight junction | 0.00478618 | 9 | MYH1A,GNAI2,CDC42,ACTB,MYH11,  GNAI3,AFDN,TJP2,EPB41L3 |
|  | gga05132 | Salmonella infection | 0.0094602 | 6 | RAB7A,ROCK2,CDC42,ARPC1A,ACTB,FLNB |
|  | gga03022 | Basal transcription factors | 0.01928293 | 4 | TAF7L,ENSGALG00000002399,TBP,GTF2B |
|  | gga04810 | Regulation of actin cytoskeleton | 0.02006432 | 10 | PIKFYVE,PPP1R12A,CDC42,ACTB,VCL,ITA3,ROCK2,ITGA6 |
|  | gga04920 | Adipocytokine signaling pathway | 0.02984347 | 5 | ACSBG2,NPY,PPKAG2,ACSL1,AGRP |
| pituitary | gga00100 | Steroid biosynthesis | 6.05E-07 | 9 | MSMO1,LSS,FDFT1,DHCR7,CYP51A1,DHCR24,ENSGALG00000048205,SQLE,LIPA |
|  | gga04510 | Focal adhesion | 0.00986934 | 16 | RHOA,COL1A1,MYLK,ITGA8,MYL2,COL4A2,CAV1,PIK3R1,PPP1R12A,MAP2K1,MYL12A,VWF,COL1A2,ZYX,PARVG,ROCK2 |
|  | gga04810 | Regulation of actin cytoskeleton | 0.02982972 | 14 | RHOA,PIKFYVE,MYLK,ITGA8,MYL2,ARHGEF7,PPP1R12A,MAP2K1,GNG12,ARPC1A,MYL12A,PIK3R1,ABI2,ROCK2 |
|  | gga04512 | ECM-receptor interaction | 0.04990265 | 7 | COL1A1,COL4A2,SDC1,ITGA8,VWF,COL1A2,HSPG2 |
| ovary | gga03010 | Ribosome | 2.27E-23 | 60 | RPL24,RPS27,RPS17,RPS15A,RPL32,RPL17,RPL5,RPL8,RPS4X,RPSAP58,RPS28,ENSGALG00000015617,RPS10,RPL27A,RPL9,RPS3A,RPS20,RPL22,RPS29,RPS16,RPL13,RPL7A,RPS3,RPL34,RPL14,RPL11,RPL3,RPS6,RPS13,RPL15,RPL12,RPL31,RPL6,RPS14,RPL36AL,RPL21,RPS7,RPL26L1,RPS12,RPL35,MRPS9,RPL35A,RPS24,ENSGALG00000007699,RPL4,RPL36,RPL19,RPL10A,UBA52,RPS15,RPL27,RPS21,RPS2,RPL37,MRPS16,RPS11,RPS26,RPL37A,RPL39,RPL29, |
|  | gga04512 | ECM-receptor interaction | 2.35E-08 | 26 | COL1A2,ITGA8,ITGA1,VTN,THBS1,HSPG2,TNC,CD44,COL6A3,ITGB5,COL4A1,COL1A1,ENSGALG00000019761,COL5A1,FN1,CD36,COL4A2,LAMA4,COL5A2,COL6A2,RELN,  ENSGALG00000003283,VWF,COL3A1,  COL6A1,ITGAV |
|  | gga04510 | Focal adhesion | 2.39E-07 | 41 | COL1A2,PDGFRA,ITGA8,ITGA1,ITGB5,VTN,  PARVG,THBS1,TNC,FLNB,RHOA,FYN,COL6A3,PTEN,COL4A1,COL1A1,MYL9,ACTN1,  ENSGALG00000019761,PDGFD,COL5A1,  FN1,PXN,PPP1R12A,COL4A2,FLNA,VCL,CAPN2,LAMA4,ENSGALG00000003283,  MYLK,COL5A2,COL6A2,RELN,ENSGALG00000007646,PPP1R12B,CAV1,VWF,COL3A1,COL6A1,ITGAV |
|  | gga04145 | Phagosome | 5.48E-05 | 27 | C3,RAB7A,CD36,THBS1,ENSGALG00000000461,CYBB,BF1,BLB2,ENSGALG00000003283,ITGB5,SFTPA2,PIKFYVE,MRC1, TLR4,BF2,NCF4,DMB2,CTSS,DYNC1LI2,  LAMP2,ENSGALG00000039554,ITGB2, MMR1L4,BLB1,C1R,ATP6V0D2,ITGAV |
|  | gga04514 | Cell adhesion molecules (CAMs) | 0.00067168 | 22 | ICOSLG,ITGA8,CADM1,ENSGALG00000031794,ENSGALG00000005257,BF1,BLB2,PTPRC,ENSGALG00000015032,ENSGALG00000002643,ENSGALG00000031430,VCAN,BF2,DMB2,PECAM1,NCAM2,ITGB2,BLB1,  CDH5,CD99,ITGAV,ENSGALG00000051068 |
|  | gga04142 | Lysosome | 0.01611466 | 17 | ABCA2,CLTA,LGMN,CTSA,AP1S3,ATP6V0D2,ASAH1,CTSC,CTSK,DNASE2B,CTSS,CTSB,LAPTM5,CTSH,LIPA,LAMP2,CLTCL1 |
|  | gga04270 | Vascular smooth muscle contraction | 0.01820782 | 16 | RHOA,RAMP2,MYLK,GNAQ,PPP1R12B,MRVI1,PPP1R12A,CO6,MYL9,EDNRA,CALD1,  ACTG2,kcnma1,PLA2G4A,ENSGALG00000037869,ACTA2 |
|  | gga00140 | Steroid hormone biosynthesis | 0.04247828 | 7 | CYP19A1,CYP11A1,STS,CYP21,COMT,  CYP17A1,HSD3B1 |
|  | gga00770 | Pantothenate and CoA biosynthesis | 0.0470073 | 4 | PANK3,DPYS,VNN1,ENPP3 |
