# Supplementary material for: Novel Regulatory Factors in the Hypothalamic-Pituitary-Ovarian Axis of Hens at Four Developmental Stages
Source: Front Genet. 2020 Nov 4;11:591672. doi: 10.3389/fgene.2020.591672 (PMC7672196; doi:10.3389/fgene.2020.591672)
Supplement: Supplementary Table 5 — The list of gene primers sequencing used for qRT-PCR validation. [file Table_5.DOCX]

TableS5. The list of gene primers sequencing used for qRT-PCR validation

Gene GenBank number Primer Sequence of nucleotide (5´-3´) Product length (bp)

*CCK* NM_001001741.1 F 5´- GGAAGGAAGGAGGAAGCGATG -3´ 295

R 5´- CAGAGAACCTCCCAGTGGAAC -3´

*LRR1* XM_421455.6 F 5´-GACAACGTGGAGCGGTTCT -3´ 289

R 5´-GGTGTTCGAGGGAATAGGGG -3´

*PLBD1* XM_015289354.2  F 5´- TGTTGAGCAGATCCCAACCC -3´ 216

R 5´- ATCGTGCTGGTAGTCTTGCC -3´

*CPLX1*  XM_424869.6 F 5´- AAAGGCAGCCGCTTTTTCTG -3´ 206

R 5´-CACGGGAACTTTTGTGCTGG -3´

*FTH1* NM_205086.1 F 5´- GTCAACAGTGCTTGGACGGA -3´ 258

R 5´- TTCAGAGCCACATCATCCCG -3´

*CYP19A1* NM_001001761.3 F 5´- GGCCTCCAGCAGGTTGAAAG -3´ 214

R 5´- ATAGGCACTGTGGCAACTGG -3´

*β-actin*  NM_205518.1 F 5´-CAGCCAGCCATGGATGATGA-3´ 147

R 5´-ACCAACCATCACACCCTGAT-3´
